# SCExV: a webtool for the analysis and visualisation of single cell qRT-PCR data

**DOI:** 10.1186/s12859-015-0757-z

**Published:** 2015-10-05

**Authors:** Stefan Lang, Amol Ugale, Eva Erlandsson, Göran Karlsson, David Bryder, Shamit Soneji

**Affiliations:** Division of Molecular Hematology, BMC B12, Lund University, Sölvegatan 19, Lund, 22184 Sweden; Lund Stem Cell Center, Lund University, Lund, 22184 Sweden

**Keywords:** Gene expression, Single cells, qRT-PCR, Webtool, FACS, Index sorting, Visualisation

## Abstract

**Background:**

Single cell gene expression assays have become a powerful tool with which to dissect heterogeneous populations. While methods and software exist to interrogate such data, what has been lacking is a unified solution combining analysis and visualisation which is also accessible and intuitive for use by non-bioinformaticians, as well as bioinformaticians.

**Results:**

We present the Single cell expression visualiser (SCExV), a webtool developed to expedite the analysis of single cell qRT-PCR data. SCExV is able to take any data matrix of Ct values as an input, but can handle files exported by the Fluidigm Biomark platform directly. In addition, SCExV also accepts and automatically integrates cell surface marker intensity values which are measured during index sorting. This allows the user to directly visualise relationships between a single cell gene expression profile and the immunophenotype of the interrogated cell.

**Conclusions:**

SCExV is a freely available webtool created to import, filter, analyse, and visualise single cell gene expression data whilst being able to simultaneously consider cellular immunophenotype. SCExV is designed to be intuitive to use whilst maintaining advanced functionality and flexibility in how analyses are performed.

## Background

The evaluation of gene expression at the single cell level can be used for a variety of applications in cell biology, including gene network reconstruction [1, 2] and the study of cell populations too rare to assay using bulk-population based approaches [3]. However, one of the most effective uses of such approaches has been to dissect cellular populations initially thought to be homogeneous, thereby revealing subgroups of cells with different functional properties [4]. The most common approach is to perform qRT-PCR on defined panels of genes, and in this respect the Biomark Fluidigm is currently the most used platform where typically 96 genes can be probed simultaneously within an individual cell. We have produced the Single Cell expression Visualiser (SCExV) a webtool specifically designed to enable non-bioinformaticians to analyse single cell gene expression data in an easy and intuitive manner. SCExV also allows the import of index cell sorting data which are the intensities of surface marker expression that are recording during the sorting process. In this way, subgroups found within a cohort of cells can be directly related back to their immunophenotype in an unbiased manner. This has great utility, for instance when the objective of the experiment is to define new cell sorting strategies or confirm existing ones.

## Implementation

SCExV is implemented using the Perl Catalyst framework and Javascript. Calculation and plots are generated using R and Bioconductor packages and called by Perl. Rotatable 3D plots are implemented in WebGL. The challenge while developing SCExV was to maintain usability whilst also providing flexibility in how analyses are performed and displayed. Therfore, some of the functions native to R/Bioconductor had to be modified to ensure cross-compatibility with each other, namely the heatmap.2 and vioplot functions.

SCExV has two main modules 1) data upload, quality control, and normalisation, and 2) analysis and visualisation. SCExV will import data from the Biomark Fluidigm platform when exported in either table or heatmap format, as well as index cell sorting data (if available) in tab-delimited text. For other platforms, SCExV accepts a simple *N* cells by *G* genes matrix of Ct values in tab-delimited text format. In order to link the two data types, SCExV uses the well ID is used as a common identifier to mark the same cell in both files. SCExV can take multiple runs and automatically concatenate them to form a single experiment.

### QC, filtering, and normalisation

At the first stage of an analysis the user has the option to remove problematic cells. This is done by choose a panel of positive control genes which SCExV will histogram. Super-imposed on these are density plots for individual chips, allowing the user to determine whether any replicates are technical outliers worthy of further scrutiny or removal. Cells are removed from the experiment if they do not fulfil user-defined criteria; for example, if control genes are expressed below user-defined threshold levels. Once initial filtering has been done, SCExV inverts the data so that for a given expression value *v*, *v*^*inverted*^=*LOD*−*v*,where *LOD* is the limit of detection representing the maximum number of PCR cycles run. Expression profiles are subsequently z-transformed [5]. The normalisation of single cell expression data is still a contentious issue as house-keeping genes are not a reliable baseline at the single cell level, regardless, we have provided several options including one that scales the Ct value of all genes within a cell to the median of a panel of house-keeping genes defined by the user. These can be used at the user’s discretion.

### Analysis

**Fig. 1:**
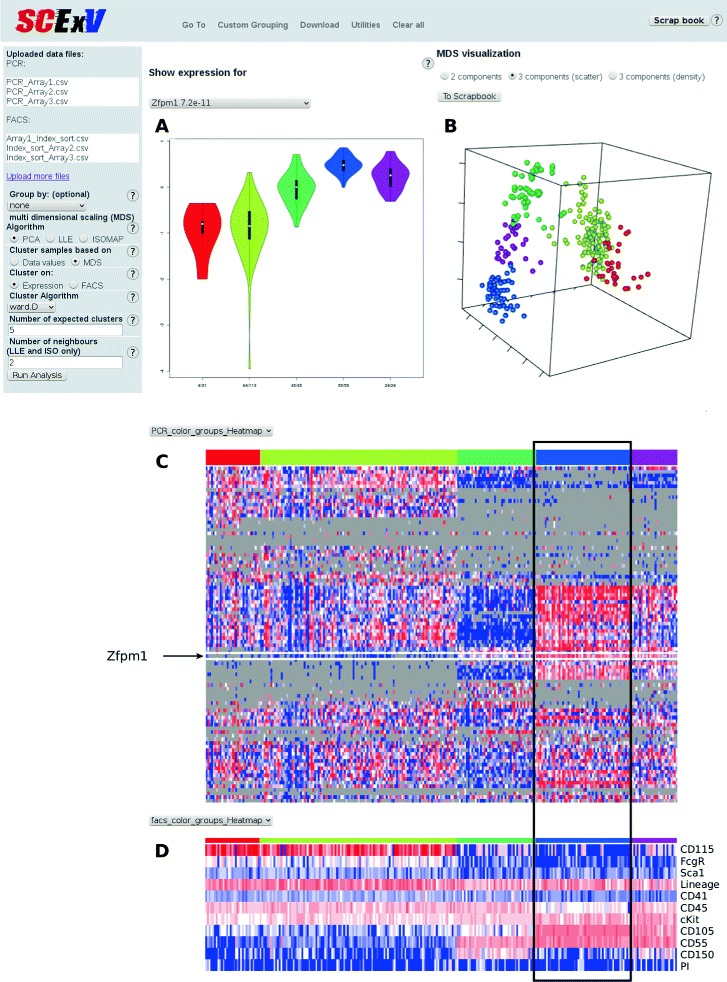
An SCExV session. Single cell qRT-PCR data has been clustered and partitioned into 5 groups (coloured bar in **c**) which defines the order of the index cell sorting data (**d**) and the colouring of cells in the PCA plot (**b**). The violin plot (**a**) gives an overview of expression within these 5 groups for a gene of interest, here *Zfpm1* which shows strong expression in the blue cluster (**a** and **c**) which denotes a cluster of cells expressing an erythroid program

The output from the analysis module is split into three main sections. The first pane shows the expression level of any selected gene within groups (e.g. clusters) as a violin plot (Fig. 1a), and the second displays the output from multidimensional scaling (PCA is shown in Fig. 1b). We have provided three viewing options i) the first 2 components ii) rotatable plot of components 1–3, and iii) 3D densities of components 1–3. Below the violin and MDS plots are heatmaps of the qRT-PCR expression data and surface marker intensities from the index sorting (Fig. 1c and 1d). Along with PCA, we have also implemented isomaps and local loop embedding (LLE) as alternatives [6]. We have provided two clustering methods; hierarchical clustering which uses the correlation distance by default (users have the option to choose the agglomeration rule), and kmeans. These can be applied to the expression and index sorting data.

### Creating/managing cell groups

The colouring scheme within heatmaps/violin/MDS plots denote groups of cells. Initially the groups are defined according to their plate (plate ID). New groups can then be defined, for example, by clustering to create groups of cells with similar expression patterns (see Fig. 1). We have provided two more ways to create groups that we call 1D/2D grouping. In 1D grouping the user has the option to chose a gene, and based on the expression level of that gene, bin the cells into as many partitions as required by providing cut-offs (e.g. low/high expressing). 2D grouping allows the user to select two genes which are plotted against each other, and groups are defined by dragging a box around the required cells (for example high/high, high/low expressing). Once confirmed, the user is returned to the analysis page where the colours in all plots are updated accordingly.

In some cases it is desirable to merge groups, for example, if two or more clusters are very similar, or several groups are composed of cells from the same sorting strategy. We have provided a fast way to do this where the groups that require merging are simply dragged together. At the same time, the order of the groups presented in the heatmaps and violin plots can also be changed. This is particularly useful, for example, where the groups of cells represent some developmental progression and the plots need to be adjusted to reflect time.

### Saving analyses

We have implemented a fast and novel method to save results during an SCExV session which we call the “scrapbook”. As figures are generated by SCExV, the user can drag and drop the image into the scrapbook (or using a dedicated button for 3D plots) which opens a new tab in the browser. A dialogue box for free text is presented, inviting the user to annotate the plot. Once confirmed, the plot and annotation is added to the scrapbook for that session. In the interests of reproducibility, we also provide a log in the scrapbook detailing which analysis steps have been executed during the current session. The number of entries to the scrapbook is unlimited for any SCExV session.

The current state of an SCExV session can be downloaded as a zip file which contains all data, the scrapbook, and R scripts used during the analysis process. This zip file can also be uploaded to SCExV to continue a session. No data is saved on the server.

## Results

This section presents an example study using data from murine myeloid-erythroid progenitor cells generated specifically to demonstrate the utility of SCExV.

### Example analysis of murine blood progenitor cells

282 murine myeloid-erythroid progenitor cells were sorted as Lineage negative/low, cKit positive and Sca1 negative and stained for a further 5 surface markers, the intensities for which were recorded during index sorting for each cell (along with scatter profiles for each cell). Two surface markers CD115 and CD55 were chosen for their potential to mark myeloid and erythroid cells respectively [7]. For each cell the expression level of 96 genes were measured by qRT-PCR using the Fluidigm Biomark platform. Cells not expressing control genes were removed, and the remaining cells were clustered on their gene expression values and partitioned into 5 groups (coloured bar in Fig. 1c). The violin plot (Fig. 1a) shows the expression of a known erythroid regulator *Zfpm1* which can be seen to have highest expression in the blue cluster. In the corresponding heatmap of cell surface markers (Fig. 1d) the blue cluster contains cells expressing CD55 but not CD115, confirming CD55 as an erythroid-associated marker. Conversely, CD115 was highly expressed on cells in the red and yellow clusters, which mark myeloid cells that are notably absent for CD55. The PCA plot coloured accordingly shows a clear division between erythroid and myeloid affiliated cells. Megakaryocyte/erythroid cells (MegE) show as a distinct group marked by CD150 (green cluster).

## Discussion

From the example we present, a potential downstream analysis would be to sort CD55 positive cells with a panel of previously known erythroid affiliated markers. From here one could determine how much overlap CD55 has with these, and indeed whether CD55 could replace two or more of them, thereby freeing channels on the cell sorter that could be used for other purposes. Alternatively, the CD55 positive subset might represent a unique subset of erythroid cells.

## Conclusions

SCExV is a user-friendly webtool that allows one to rapidly perform advanced analyses of single cell expression data without the need for programming skills. The ability to concurrently analyse index cell sorting data greatly enhances the utility of SCExV as it enables cell sorting strategies to be designed/confirmed/adjusted, which in turn promotes a faster development of protocols aimed at dissecting cellular heterogeneity.

## Availability and requirements

**Project name**: SCExV**Project homepage**: http://stemsysbio.bmc.lu.se/SCExV/ where tutorial videos and example data are also provided.**Programming language**: Perl Catalyst, Javascript, R.**Browser requirements**: OpenGL enabled browsers. Currently optimised for Chrome and Firefox.**License**: GPLv3**Any restrictions to use by non-academics**: No
